# Restoring Visual Acuity in Dynamic Conditions with a Vestibular Implant

**DOI:** 10.3389/fnins.2016.00577

**Published:** 2016-12-22

**Authors:** Nils Guinand, Raymond Van de Berg, Samuel Cavuscens, Robert Stokroos, Maurizio Ranieri, Marco Pelizzone, Herman Kingma, Jean-Philippe Guyot, Angélica Pérez Fornos

**Affiliations:** ^1^Service of Otorhinolaryngology Head and Neck Surgery, Department of Clinical Neurosciences, Geneva University HospitalsGeneva, Switzerland; ^2^Division of Balance Disorders, Department of ENT, Maastricht University Medical CentreMaastricht, Netherlands; ^3^Faculty of Physics, National Research Tomsk State UniversityTomsk, Russia

**Keywords:** bilateral vestibular loss, vestibular implant, dynamic visual acuity, vestibulo-ocular reflex, electrical stimulation, cochlear implant

## Abstract

Vestibular implants are devices designed to rehabilitate patients with a bilateral vestibular loss (BVL). These patients lack a properly functioning vestibulo-ocular reflex (VOR), which impairs gaze stabilization abilities and results in an abnormal loss of visual acuity (VA) in dynamic situations (i.e., severely limiting the patient's ability to read signs or recognize faces while walking). We previously demonstrated that the VOR can be artificially restored in a group of BVL patients fitted with a prototype vestibular implant. This study was designed to investigate whether these promising results could be translated to a close-to-reality task, significantly improving VA abilities while walking. Six BVL patients previously implanted with a vestibular implant prototype participated in the experiments. VA was determined using Sloan letters displayed on a computer screen, in four conditions: (1) with the patient standing still without moving (static), (2) while the patient was walking on a treadmill at constant speed with the vestibular implant prototype turned off (systemOFF), (3) while the patient was walking on a treadmill at constant speed with the vestibular implant prototype turned on providing coherent motion information (systemON_motion_), and (4) a “placebo” condition where the patient was walking on a treadmill at constant speed with the vestibular implant prototype turned on providing reversed motion information (systemON_sham_). The analysis (one-way repeated measures analysis of variance) revealed a statistically significant effect of the test condition [*F*_(3, 12)_ = 30.5, *p* < 0.001]. Significant decreases in VA were observed with the systemOFF condition when compared to the static condition (Tukey *post-hoc p* < 0.001). When the vestibular implant was turned on, delivering pertinent motion information (systemON_motion_) the VA improved to close to normal values. The improvement disappeared in the placebo condition (systemON_sham_) and VA-values also dropped significantly in this condition (Tukey *post-hoc p* < 0.001). These results are a significant step forward in the field, demonstrating for the first time in humans that gaze stabilization abilities can be restored with a vestibular implant prototype. The vestibular implant shows considerable promise of being the first-ever effective therapeutic alternative for patients with a BVL in the near future.

## Introduction

The vestibular implant is a device designed to artificially restore the vestibular function using motion modulated electrical stimulation of the peripheral vestibular system. In the past two decades, several groups have demonstrated that three key aspects of vestibular function: the vestibulo-ocular reflex (VOR; Merfeld et al., 2007; Lewis et al., 2010; Dai et al., 2011; Perez Fornos et al., 2014), specific postural responses (Phillips et al., 2013), as well as vestibular percepts (Guinand et al., 2015) can be artificially elicited and restored using a vestibular implant. This confirms that it is possible to effectively transmit motion information to the central nervous with such a device. The vestibular implant concept could be applicable in cases of severe bilateral loss of the vestibular function (BVL), a very disabling and poorly recognized condition for which no effective treatment exists (Guinand et al., 2012a; Sun et al., 2014). Our group has recently demonstrated partial restoration of the VOR in a group of BVL patients fitted with a prototype vestibular implant, using motion modulated electrical stimulation of the vestibular nerve (Perez Fornos et al., 2014). In certain cases the velocity of the elicited eye movements was within the range of compensatory eye movements observed in healthy subjects during walking or running (Grossman et al., 1989; Guinand et al., 2015) and the electrically evoked VOR displayed a similar frequency response as the physiological VOR (Van de Berg et al., 2015). These results can be considered as a milestone in the development of a vestibular implant, confirming in humans pioneering findings obtained in animal research (Gong and Merfeld, 2002; Dai et al., 2011).

The next fundamental question was whether this artificial vestibular reflex could be useful to improve performance on a clinically significant, more complex task. The majority of patients diagnosed with a BVL describe a clinical manifestation consisting of blurred vision or oscillopsia. For example, they experience difficulties recognizing faces or reading signs while walking. This is mainly attributed to the loss of the vestibular reflexes, in particular of the VOR that holds a crucial role in the mechanism of gaze stabilization. In the clinic, this can be quantified as a pathological drop of visual acuity (VA) in dynamic conditions compared to a static condition (Guinand et al., 2012b). Some testing protocols use passive, unpredictable high velocity yaw or pitch head movements as stimuli for the dynamic condition. They have demonstrated high sensitivity in revealing a BVL (Schubert et al., 2006; Vital et al., 2010). However, a drawback of these methods is that the stimuli used are not physiological. Another more representative method of the everyday challenge faced by BVL patients is the evaluation of VA while walking on a treadmill at controlled velocities (Lambert et al., 2010). This original method has been shown to be reliable and sensitive to detect BVL (Hillman et al., 1999), even at low walking speeds of 2 km/h (Guinand et al., 2012b).

Demonstrating the restoration of gaze stabilization abilities in BVL patients, particularly in a close-to-reality task would constitute a significant step forward in the rehabilitation of vestibular deficits. In the present study, we investigated whether motion-modulated electrical stimulation could be used to normalize VA abilities while walking in a group of BVL patients chronically implanted with a vestibular implant.

## Materials and methods

### Patients and device

Twelve BVL patients, unilaterally or bilaterally deaf, were recruited at the Service of Otorhinolaryngology and Head and Neck Surgery at the Geneva University Hospitals and at the Division of Balance Disorders at the Maastricht University Medical Center. Strict inclusion criteria were implemented, and have been described in detail previously (Guinand et al., 2015).

Patients were fitted with prototype vestibular implants. These devices consisted of a modified cochlear implant (MED-EL, Innsbruck, Austria) that provided, in addition to the cochlear array, extra-cochlear electrodes for “vestibular” stimulation. Extra- or intra-labyrinthine implantation of the electrodes in the vicinity of the ampullary branches of the vestibular nerve, as previously described (Kos et al., 2006; Van de Berg et al., 2012), was performed. The vestibular implant was activated no earlier than 3 weeks after surgery. Six out of the twelve implanted patients were available for dynamic VA experiments presented in this paper (Table 1).

**Table 1:** **Demographics and implantation details of participating patients**.

**Patient**	**Sex**	**Etiology**	**Age (at implantation)**	**Implantation year**	**Active electrode**	**Surgical approach**	**Baseline stimulation amplitude (Dynamic range; μA)**
S1	M	Idiopathic	68	2007	PAN	EL	360 (170)
S2	M	Congenital/idiopathic	46	2008	PAN	EL	300 (100)
S3	F	Traumatic	67	2013	SAN	IL	410 (300)
S4	F	Meningitis	48	2012	SAN	IL	200 (180)
S5	M	DFNA9	66	2013	PAN	IL	120 (80)
S6	M	Traumatic	53	2015	SAN	IL	350 (450)

*PAN, posterior ampullary nerve; EL, extra-labyrinthine; SAN, superior ampullary nerve; IL, intra-labyrinthine*.

A regular cochlear implant processor (Tempo+MED-EL, Innsbruck, Austria) was used to control the electrical stimulation delivered by the selected electrode using a customized transformation unit connected to the auxiliary input of the processor (Pelizzone et al., 2013). Angular head motion was captured with a three-axis gyroscope (device based on the sensor LYPR540AH; ST Micro-electronics; Geneva, Switzerland), fixed to the patient's head using a customized helmet.

### Electrical stimulation

As the predominant components of head movements during walking are pitch and vertical translation (Grossman et al., 1989), electrodes in the vicinity of the posterior (PAN) or superior (SAN) ampullary nerves were selected to deliver motion information using electrical currents. Theoretically, stimulation of these vertical vestibular nerve branches should generate vertical compensatory eye movements (Suzuki et al., 1964). Only one vestibular electrode was active during the experiments and all cochlear electrodes were turned off. As already described in previous publications, to generate bidirectional eye movements (i.e., upwards and downwards when stimulating the vertical nerve branches) when using unilateral vestibular stimulation, it was necessary to first restore and maintain a *baseline stimulation* of the vestibular nerve (Guyot et al., 2011; Perez Fornos et al., 2014; Guinand et al., 2015). In this study, we chose a supraphysiological baseline stimulation profile consisting of trains of biphasic, charge-balanced (200 μs/phase) pulses presented at a rate of 400 pulses per s. These stimulation parameters were selected because they have proved to be particularly effective for activating the vestibular system in our particular setting. The amplitude of the baseline stimulation was set in the middle of the dynamic range measured for each patient (Guinand et al., 2015). Once in the *adapted* state (Guyot et al., 2011), the motion signal captured by the head mounted gyroscope could be used to up- and down-modulate the amplitude of the train of pulses delivered via the SAN or PAN vestibular electrodes.

We arbitrarily chose to implement a simple linear transfer function between measured pitch head velocity and electrical stimulation delivered via the SAN or PAN electrode. It was defined based on the previously measured dynamic range and eye movement response characterized for each subject (Guinand et al., 2015). A maximum of 85% of each patient's dynamic range was used to code for 30°/s, based on previous data on the main characteristics of the VOR during locomotion (Grossman et al., 1989). For safety reasons, maximum stimulation delivered was hard coded to be limited to 90% of the patient's dynamic range, to avoid excessively high currents in case any abrupt, rapid head movement occurred.

### Visual acuity measurements

During the experiments, patients had to read aloud sequences of Sloan optotypes (CDHKNORSVZ) of decreasing size displayed in a random order one at a time on a computer screen (15 inches). The screen was positioned at eyes' height, 2.8 m in front of the patient. The sequence started with a five letters presentation at 1 logMAR (logarithm of the Minimum Angle of Resolution). If the letter recognition rate was above chance (>10%), the letter size was decreased by a step of 0.1 logMAR and five new letters were presented one at a time. The same procedure was continued until the recognition rate for a given letter size dropped below chance (≤10%). Two almost identical additional runs were repeated.

The experiments were carried out on a treadmill in four conditions: (1) with the patient standing still (static), (2) while the patient was walking at a constant speed with the vestibular implant turned off (systemOFF), (3) while the patient was walking at a constant speed with the vestibular implant turned on and delivering *coherent* motion information to the patient's vestibular nerve (i.e., amplitude of the baseline stimulation modulated using the signal coming from the pitch axis of the gyroscope; systemON_motion_), and (4) while the patient was walking at a constant speed with the vestibular implant turned on and delivering *incoherent* motion information to the patient's vestibular nerve (i.e., amplitude of the baseline stimulation modulated using the reversed signal coming from the pitch axis of the gyroscope; systemON_sham_). Walking speed was set between 2 and 4 km/h, at the maximum where the patient felt safe and could walk without holding the handrails in the systemOFF condition. Once the maximum safe speed for the patient was selected, it was kept constant for all the dynamic conditions. The order in which each of the three dynamic conditions was conducted was determined using a Latin square design, randomized across patients. All experiments were written in MATLAB (R2010a; Mathworks, Natick MA, USA) using the Psychtoolbox (Brainard, 1997; Pelli, 1997; Kleiner et al., 2007).

Raw data, expressed in logMAR, were converted to decimal VA-values and normalized to VA obtained in static conditions (Holladay, 1997). A one-way repeated measures analysis of variance (ANOVA) was conducted to compare VA across conditions. All statistical analyses were performed with the statistics package for SigmaPlot 13.0 (Systat Software, Inc., Chicago, IL, USA).

### Ethics statement

All subjects gave written informed consent in accordance with the Declaration of Helsinki. Approval of the ethical committees of the Geneva University Hospitals (NAC 11-080) and of the Maastricht University Medical Center (NL36777.068.11/METC 11-2-031) was obtained.

## Results

All patients were able to complete the procedure at their own maximum safe walking velocity (2–4 km/h). Absolute VA-values obtained in each condition are presented in Table 2. Please note that lower logMAR-values indicate better scores.

**Table 2:** **Absolute VA-values obtained in each condition per patient [logMAR]**.

**Patient**	**Static VA**	**SystemOFF**	**SystemON_motion_**	**SystemON_sham_**	**MSWV**
S1	0.04	0.18	0.04	0.14	2
S2	−0.07	0.19	0.04	0.13	4
S3	−0.21	0.07	−0.14	−0.02	3
S4	−0.34	−0.17	−0.31	−0.31	4
S5	−0.13	0.00	−0.11	−0.05	4
S6	0.06	0.31	0.22	0.33	3

*Maximum safe walking velocities [MSWV (km/h)] in each case are also indicated*.

Compared to the static condition, all six patients experienced a drop in VA while walking on the treadmill in the systemOFF condition, ranging from 0.13 to 0.28 logMAR in absolute value (a loss of 0.1 logMAR corresponds to a loss of one line on a standard letter chart used for the measurement of the VA). In the systemON_motion_ condition, the VA improved in all patients compared to the systemOFF condition, and even equaled the value of the static condition in one patient (S1). The VA differences between the static and the systemON_motion_ conditions ranged from 0 to 0.16 logMAR. The VA differences between the static and the systemON_motion_ conditions were smaller than the VA differences between the static and the systemON_sham_ conditions in all six patients. The VA differences between the static and the systemON_sham_ conditions were smaller than the VA difference between the static and the systemOFF conditions, except for S6. The range of the VA differences between the static and the systemON_sham_ condition was 0.03 to 0.28 logMAR.

**Figure 1:**
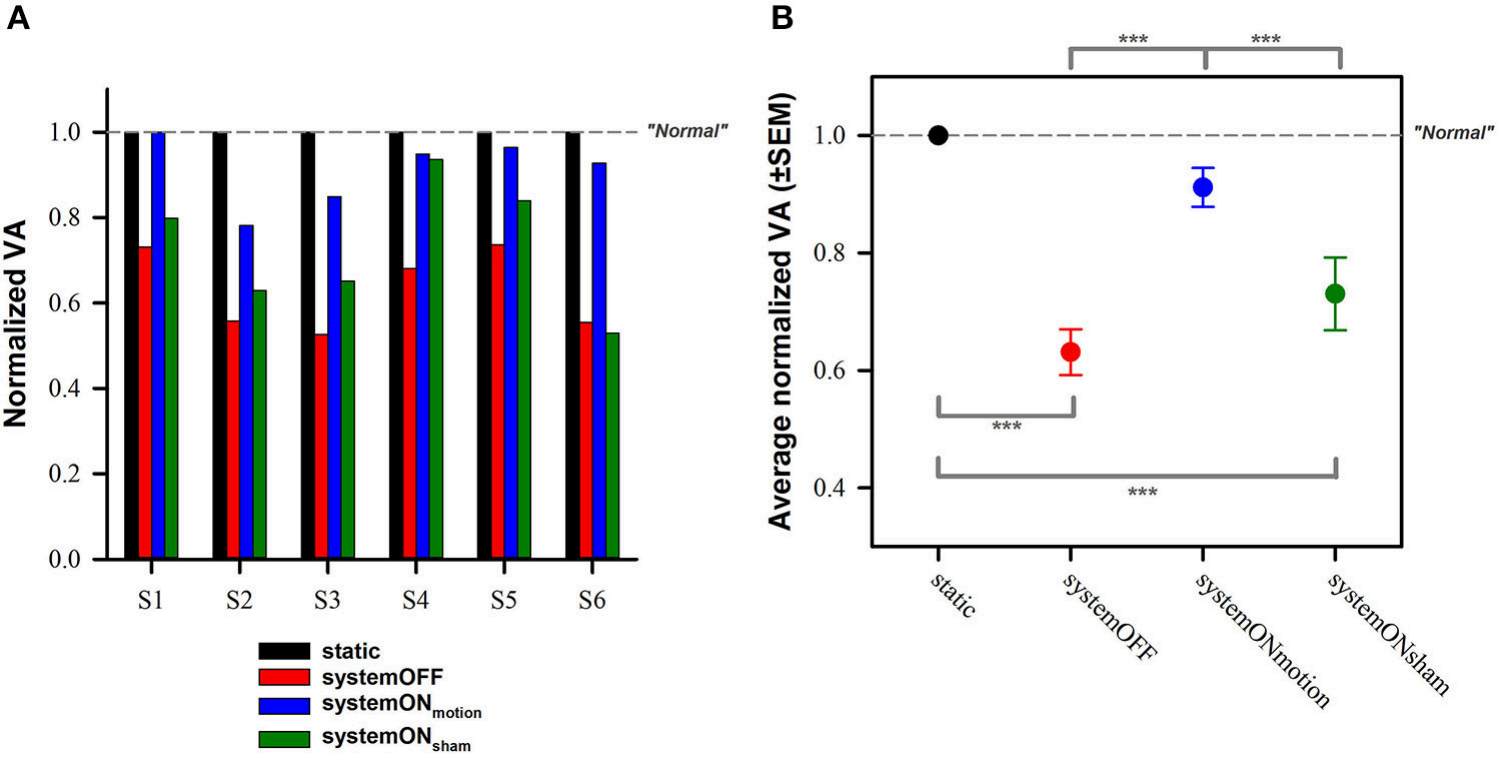
**Normalized visual acuity results. (A)** Individual results obtained in the dynamic conditions (colored bars; red—systemOFF, blue—systemON_motion_, green—systemON_sham_) for the six participating subjects, normalized to results obtained in the static condition (black bar). **(B)** Average normalized results (± standard error of the mean, *SEM*). ^***^Indicates significant differences between conditions in the *post-hoc* tests (Tukey). Dotted gray lines indicate theoretical performance of “normal” subjects (i.e., no loss of visual acuity in dynamic situations).

Normalization of individual VA scores to values obtained in the static condition allows a better representation and facilitates comparison of the previously mentioned trends for each patient (Figure 1A). The ANOVA analysis showed a statistically significant difference between conditions [*F*_(3, 12)_ = 30.49, *p* < 0.001]. *Post-hoc* tests (Tukey) revealed a significant (*p* < 0.001) increase of the VA loss in the system OFF and in the systemON_sham_ conditions, compared to the static and the systemON_motion_ conditions. No significant differences were found, either between the static and the systemON_motion_ conditions, or between the system OFF and the systemON_sham_ conditions (see Figure 1B).

## Discussion

These results clearly indicate that the vestibular implant successfully transfers motion information to the brain, leading to restoration of VA abilities in a dynamic situation (walking in standardized conditions). This represents the first demonstration of functional rehabilitation using the concept of motion-modulated electrical stimulation of the vestibular nerve in humans, and therefore constitutes a fundamental milestone in the field.

To investigate whether it was possible to improve gaze stabilization abilities in BVL patients wearing a prototype vestibular implant, a protocol representative of one of the most common complaints was implemented. When walking, patients with a BVL present a significant loss of VA which is correlated with the presence of oscillopsia (Guinand et al., 2012b). Using motion modulated electrical stimulation, the VA measured in dynamic conditions (i.e., while walking) was significantly improved to a value close to that measured in static conditions in all six patients tested. The fact that the VA improvement decreased significantly in the systemON_sham_ condition further confirms that observed improvements were due to the properly functioning vestibular implant. Interestingly however, the VA loss observed in the systemON_sham_ condition was slightly smaller than that observed in the systemOFF condition (non-significant), suggesting that some useful motion information could still have been extracted by the brain in the systemON_sham_ condition where the gain was reversed.

It is generally accepted that the drop of VA measured in dynamic conditions in patients suffering from a BVL is due to a poorly functioning (or absent) VOR, which is generally considered the main vestibular mechanism involved in gaze stabilization. Initially, we wanted to quantify the artificially generated VOR during the VA task in dynamic conditions in order to demonstrate that any measured improvements would be due to the restoration of this reflex with our vestibular implant prototype. We attempted recording eye movements while we measured the VA in dynamic conditions using a fast 2D video-oculography system (EyeSeeCam VOG; Munich, Germany), but were not successful in achieving precise recordings. In order to avoid artifacts due to goggle slippage, the goggles had to be very tightly adjusted. This was too painful to patients after just a few minutes, not giving enough time to complete the task. Furthermore, the tightly fixed glasses also disrupted the visual abilities of patients, especially at near-threshold values. As a consequence, we decided not to record eye movements during VA measurements. However, in an attempt to better understand how the magnitude of the electrically evoked VOR influenced the VA results we decided to compare the latter with previously presented results of the artificial VOR measured in static conditions (Guinand et al., 2015). Surprisingly, we found no correlation between the magnitude of the evoked VOR and the observed improvements in the systemON_motion_ condition. This could of course be due to the small sample size of the study. However, it could also suggest other vestibular mechanisms could also be substantially contributing to gaze stabilization. A first hypothesis is that, by electrically delivering motion information to the vestibular nerve, other vestibular reflexes are also activated. Indeed, although it was not systematically documented in this study, during the static artificial VOR measurements we observed that in some cases sinusoidal head movements were evoked in parallel to eye movements. Moreover, these head movements were phase locked with the sinusoidal electrical stimulus. This strongly suggests that the vestibulo-collic and the vestibulo-spinal pathways were also activated during our experiments. This hypothesis is supported by observations of other research teams. For example, postural responses have been reported upon electrical stimulation of the ampullas (Phillips et al., 2013) and even by using motion modulated stimulation delivered by intracochlear electrodes of a regular cochlear implant (Cushing et al., 2012). More recently, direct activation of vestibular reflexes upon electrical stimulation delivered through the intracochlear array of the cochlear implant has also been demonstrated (Parkes et al., 2016).

Future research efforts will be devoted to a more comprehensive evaluation of vestibular function, well-beyond the VOR. For example, a matter of particular interest will be to better understand whether the activation of the vestibulo-collic pathway results from current spread to the otolithic organs, or whether the role of the semicircular canals in the control of posture has been underestimated. In addition, up to now all our experiments have been carried out while activating a single electrode at a time. Future experiments we will assess the simultaneous use of multiple vestibular electrodes (in contact with all three ampullary nerves) for integration of 3D angular motion information. This will imply the development of more complex stimulation parameters and strategies. Another important aspect of future developments will involve the refinement of the electrode design and of the surgical insertion techniques to optimize electrode positioning (i.e., selectivity of the stimulation), while preserving any pre-existing auditory and residual vestibular function. This is of crucial importance as the majority of patients with a BVL have normal or only mild hearing loss. In addition, to warrant successful translation of vestibular implants to the clinic, surgical procedures should be simplified and standardized as much as possible in order to become accessible to most of otologists. Finally, a unique aspect of the vestibular implant is that it is the first experimental setup that allows activating the vestibular system exclusively, without the unwanted contribution of other sensory modalities (e.g., vision, proprioception) that intervene in the complex activities mediated by balance. We expect thus that basic research studies with this device will open new doors increasing our fundamental knowledge on the physiology of the vestibular system and its interactions with extra-vestibular mechanisms.

Finally, it is worth mentioning that the promising results presented here were obtained with a first-of-its-kind, rudimentary vestibular implant, and during acute testing sessions. Indeed, it could be expected that both improved devices and sufficient training (i.e., when patients have enough time to adapt and use the full potential of the artificial vestibular information), would result in improved performance and rehabilitation prospects. We are therefore convinced that the vestibular implant is an evolutionary device with an immense clinical and research potential. Further research and development in this field are thus justified and warranted.

## Author contributions

All authors participated in the design of the experimental protocol and analysis. NG, AP, RV, SC, and MR carried out the experiments. NG, RV, RS, and JG performed the implantations. AP, MR, and SC prepared the figures with the help of other authors. NG and AP wrote the manuscript, and all authors contributed to its editing. AP and NG supervised all aspects of the work.

## Funding

This work was funded by the European Commission (FP7 CLONS, Project 225929) and by the AURIS foundation (http://www.aurisfoundation.org).

### Conflict of interest statement

The authors disclose that MED-EL (Innsbruck, Austria) has provided financial support in other research projects as well as travel expenses for scientific meetings.
